# Do R&D intensity and capacity utilisation matter for SMEs’ innovations within the CEE region? Testing moderating roles of different ownership structures

**DOI:** 10.1371/journal.pone.0296873

**Published:** 2024-01-12

**Authors:** Raymond Darfo-Oduro, Viktor Prokop, Jan Stejskal, Viktorie Klímová, Vladimír Žítek

**Affiliations:** 1 Institute of Economic Sciences, Faculty of Economics and Administration, University of Pardubice, Pardubice, Czech Republic; 2 Science and Research Centre, Faculty of Economics and Administration, University of Pardubice, Pardubice, Czech Republic; 3 Department of Regional Economics, Faculty of Economics and Administration, Masaryk University, Brno, Czech Republic; Alexandru Ioan Cuza University: Universitatea Alexandru Ioan Cuza, ROMANIA

## Abstract

Existing innovation literature has assumed that the relationship between firms’ R&D intensity and innovation take place without the interplay of other organizational factors. However, the reality differs, and research to date has shown that other factors affecting firms’ innovation need to be considered. This is important especially in Central and Eastern Europe (CEE) countries, which are highly dependent on both internal and external R&D and are associated with an inability to use R&D resources effectively. This study therefore responds to calls for further analysis, especially within the CEE region, and focuses on the role of two factors affecting SMEs’ innovativeness and their effects, which have been mixed so far. First, we investigate the effects of SMEs’ R&D intensity and capacity utilisation on product innovation. Second, we reveal the moderating role of SMEs’ different ownership structures (ownership concentration; private/public ownership; family/non-family ownership) in the relationship between R&D intensity and product innovation. We confirm that CEE SMEs’ ownership concentration and private ownership moderate the relationship between R&D intensity and product innovation. In contrast, we reject our hypothesis expecting that family ownership of SMEs can significantly moderate the relationship between R&D intensity and product innovation. Interestingly, we also show that the relationship between capacity utilisation and innovation is non-linear (inverted U-shaped). This study makes a significant contribution in the form of analysis within the CEE region, whose innovation systems are seen to be weak, and it is therefore necessary to bring new knowledge and recommendations to managers and public policymakers.

## 1. Introduction

As the business world becomes competitive, firms are exploiting their resources and expanding their capacity to stay competitive and improve their performance [1]. One important way for firms to remain competitive is to be innovative [2]. To this end, a plethora of studies have investigated the determinants of firms’ innovation (for example [3–6]). These primarily include various R&D activities [7], (foreign) cooperation and knowledge sourcing [8] and public funding [9], among others. However, in studying the determinants of firm innovation, particular emphasis has been placed on the effect of R&D intensity that can act as a catalyst for innovation [10]. For example, Savrula and Incekarab [5] revealed that R&D intensity directly influences firm innovation in both high innovation countries such as Switzerland and Finland and countries catching up with innovation leaders, which include, for example, countries in Central and Eastern Europe (CEE; [11]. Similarly, Tsung-chun et al. [12] confirmed that R&D intensity is important for a firm’s technological efficiency, as in the case of Taiwan’s semiconductor industry.

Moreover, the simplistic assumption and omission of the important role of capacity utilisation and ownership structure in innovation activities of firms are another important limitation of prior studies, despite the fact that the literature recognises the important role of firm processes [5]. In fact, the literature indicates that, with the same level of investment in R&D, countries show different levels of innovation [5]. For example, in a study by Zdunczyk and Blenkinsopp [13], firm ownership was observed to be an important determinant of firm innovation, thereby giving credence to the importance of organizational factors in innovation performance. There is therefore a validity problem with previous studies that have assumed that R&D intensity affects firm innovation without consideration of the ownership structure or the utilisation of capacity. Earlier studies on the role of R&D intensity on innovation performance failed to recognise the importance of capacity utilisation of firms and ownership structure.

The role of ownership in fostering innovation through its interaction with R&D has been argued on two fronts. First, Beneito et al. [14] observed that ownership is the source of funding firms’ R&D. Providing financing for R&D activities is a strategic decision generally made by owners of the firm. Second, from the agency theory literature [15], the conflict between owners and managers of firms explains the differences in preference in the allocation of resources. Quite distinct from the fact that investing in R&D is an issue of resources, another important aspect is whether the owners of the firms perceive R&D, among all the competing needs, to be a priority. It has therefore been argued that owners’ sensitivity to innovation activities will partly determine R&D funding in the firm [14]. The owners’ power, usually explained by the concentration of ownership, will therefore be at play. Owners of firms who naturally have a long-term interest press for enhanced R&D activity and work to provide resources for it. The link between financiers’ ownership size in a firm and how such owners influence R&D in promoting innovation has been documented in prior studies [16]. Empirical evidence shows that firms owned and controlled by a central government invest in R&D to improve innovation performance whereas other firms owned by local governments or private and foreign owners are less responsive to R&D needs and innovation [10]. Such findings show that the ownership structure plays a role in influencing firms’ innovation, but it is not yet fully mapped out, and the results can be considered rather mixed.

Against the above literature backdrop, the main motivation of this study is to investigate the role of R&D intensity in SMEs’ innovation in the context of organizational processes, such as capacity utilisation and different ownership structures (ownership concentration; private/public ownership; family/non-family ownership) within the CEE region. More specifically, we aim to examine the role of capacity utilisation of SMEs and how different ownership structures of SMEs moderate the relationship between R&D intensity and product innovation within the CEE region, which is called, in terms of the innovation performance, an emerging European innovation system. Such a system is, among other things, specific in that it has not yet been able to fully utilise its innovative potential and has been more dependent on external knowledge, R&D, and technological procedures [17]. These procedures are adopted from abroad—quite often, Western Europe. Moreover, within the framework of emerging European innovation systems, it is possible to encounter less developed social capital and trust between cooperating partners [11], which can be partly solved by, for example, a family ownership structure characterised by higher trust between partners, thereby creating a suitable innovation climate. Thus, studying the question of different ownership structures also appears to be relevant for this region.

This study responds to the shortcomings of existing research and contributes to the current state of knowledge by integrating the almost ignored but important issues of capacity utilisation and the different ownership structures of firms into the innovation-generating process. Compared to prior research [3–5], we show that capacity utilisation and different ownership structures play a vital role in the innovation performance processes. Contrary to earlier studies in this area that assumed the traditional determinants, such as human capital, R&D intensity, and firms’ technological capabilities [18, 19], and related variables as the sole reason for changes in innovation performance, we demonstrate that increasing firms’ capacity utilisation is vital for innovation performance, but becomes inimical after a high level of capacity utilisation. To date, the literature has been split on the relationship between innovation and capacity utilisation. Some authors have suggested that operating below capacity frees up resources for innovation activities [20, 21]. Other studies have also emphasised the importance of below-capacity utilisation in innovation performance; however, these authors state that the type of slack resource is crucial [22]. A further contribution our study makes to existing literature is the introduction of different ownership structures into the innovation model. We prove, contrary to earlier studies [1, 23], that the ownership structure of a firm moderates the relationship between R&D intensity and firm innovation—a phenomenon prior studies have rather ignored, as far as we know.

The remainder of this study is organized as follows. Section 2 presents a review of the literature, hypothesis development, and the perspective of the CEE region. Section 3 then explains the research methodology. The data analysis, followed by the interpretation of the results of the data analysis, is presented in the Section 4. Finally, the results of the study are discussed and conclusions drawn.

## 2. Literature review, hypothesis development, and CEE region perspectives

### 2.1 Effects of R&D intensity on firms’ innovation

Generally speaking, a firm’s ability to absorb and assimilate new knowledge is a function of (i) its R&D activity [24] and (ii) its ability to translate knowledge generated internally and sourced externally into useful firm output [25]. Therefore, firms with higher R&D intensity are better positioned to generate innovative ideas as well as more easily assimilate and adopt external knowledge. However, despite these general statements, findings of the prior literature show varied outcomes, and the results on the effect of R&D intensity on innovation have not been consistent. From one point of view, high R&D intensity, although not a panacea for firm innovation, improves the likelihood of a firm to enhance its innovation [5, 26]. Similarly, Ivus et al. [9] revealed that increases in R&D intensity and R&D expenditures are associated with improved innovation. Yet some studies have identified inhibiting factors that limit the effects of R&D intensity on firms’ innovation. For example, Charlot et al. [27] showed that R&D intensity improves firm innovation only when R&D expenditures are above a minimum level.

Another string of studies showed the negative effects of R&D intensity on firms’ innovation. The literature reviewed by Cheng-Yu et al. [28] highlighted a mixed effect of R&D intensity on firm innovation. The authors’ findings, based on firm-level data from Japan, Germany, the UK, and the USA between 1999 and 2003, suggest that greater R&D intensity increases firms’ exploitative tendencies but reduces their explorative tendencies. This is not an isolated finding from the extant literature. Kuang et al. [29] untangled the complex relationship between R&D intensity and innovation. The authors relied on data from 257 listed Chinese high technology manufacturing firms from 2008 to 2015. Their findings revealed an inverted U-shaped relationship, indicating that technology innovation increases with R&D intensity at a decreasing rate, but technology innovation falls after R&D reached and exceeded a specific threshold. Based on the review of extant literature, the effect of R&D intensity on firm innovation is complicated.

Putting the above arguments into the context of the CEE region and its firms, the literature recognises that governments in CEE countries are making efforts to improve both the countries’ and firms’ innovation performance through R&D investments [5]. However, the literature also shows that innovation activities in CEE countries are a departure from what happens in the Western world. Prokop et al. [11] argue that firms in CEE countries adopt inward innovation activities whereas, in Western countries, collaboration has been the norm. This could partly explain why Savrula and Incekara [5] observed that CEE countries achieve very little innovation with their R&D expenditures. As such, CEE countries present a special case requiring an in-depth understanding of how R&D translates into firm innovation. It is instructive to note that, although firms in CEE countries have not been efficient in translating innovation activities to innovation outcomes, there is evidence that the economies of CEE countries are driven by innovation [17, 30].

Summing up, the evidence of R&D enhancing innovation performance of firms in CEE countries paints a complicated picture. Whereas a section of the literature argues that firm R&D in CEE countries improves firm innovation, another segment of the literature indicates that, contrary to expectations, R&D intensity is not an important source of firm innovation [29]. Therefore, in this study, we put the relationship between R&D intensity and firms’ innovation in the context of small and medium-sized enterprises (SMEs) in CEE countries, where the prior research generally shows that increasing R&D intensity positions firms for higher innovation performance. Thus, this study postulates the following hypothesis:

***H***_***1***_: *Increasing R&D intensity positively influences product innovation performance in CEE countries*.

### 2.2 Linking capacity utilisation and firms’ innovation

The link between capacity utilisation and firm innovation is an important grey area in the innovation literature. Although prior literature presents an investigation into capacity utilisation, very little is known about the relationship between capacity utilisation and innovation. Much of the literature on the relationship between capacity utilisation and innovation is old, incomplete, and scant. For example, Fevolden and Gronning [31] combined innovation and capacity utilisation in a high throughput system to highlight the tension between capacity utilisation and firm innovation in the context of mass production. According to the authors, achieving capacity utilisation through mass production requires the use of existing technologies that compromise a firm’s ability to be innovative. Thus, increasing capacity utilisation reduces the potential of firms to be innovative. In contrast with these findings, Nohria and Gulati [32] showed that the relationship between capacity utilisation and innovation is an inverted U-shaped relationship, indicating that extreme levels of capacity utilisation are inimical to firms’ innovation. A large body of dated literature has revealed that reduced capacity utilisation is a recipe for innovation [33–35]. Findings from these studies are premised on the thinking that, in the presence of lower levels of capacity utilisation, slack resources can be invested in R&D to improve innovation performance [21, 31]. Given that R&D investments are associated with some level of uncertainty, it can be argued that, when resources are stretched thin, firms will be less willing to invest in R&D than when there are slack resources. Fevolden [20] argued that lean production, a production process that minimises wastage, is an important step that frees resources for innovation activities while simultaneously increasing the utilisation of capacity. Accordingly, it is argued that, through lean production, the narrative that capacity utilisation and product innovation are not mutually compatible is changed.

Recent studies have indicated a significant effect of slack resources from low capacity utilisation on innovation. Nguyen and Trinch [36] revealed that financial resource slack is inimical to a firm’s innovation efforts, but human resource slack encourages firm innovation. These authors’ findings fuel the controversy surrounding the relationship between capacity utilisation and firm innovation. Therefore, it follows that excess capacity, in agreement with Nguyen and Trinch [36], does not automatically translate into innovation activities; the type of resource is important and what firm owners and management decide to use the excess resources for remains critical to innovation performance. Meyer and Leitner [22] used cross-sectional survey data from 250 non-profit organizations in Austria to distinguish between financial and human resource slack. The authors assessed the effect of these two types of slack on innovation and observed that human resource slack, but not financial slack, has a positive effect on innovation.

CEE firms and countries have experienced capital slack over a long period. Structural reforms in the 1990s, when CEE countries transitioned into capitalism, caused a decline in output, which resulted in an accumulation of unused capital not useable in a free market [37]. Thus, firms and states within the CEE territory, based on the argument put forward by Meyer and Leitner [22], cannot leverage such slack to improve innovation. Yet it is important to recognise that, with changing economic structures in CEE, such slack is being absorbed and could become a source of R&D activities in CEE countries [38]. Thus, the increasing use of these slack resources could potentially be channelled into innovation activities to increase firms’ product innovation performance. Therefore, the current study postulates in the context of the CEE region the following hypothesis:

***H***_***2***_: *There is an inverted U-shaped relationship between firms’ capacity utilisation and product innovation in CEE countries*.

### 2.3 Moderating roles of different ownership structures

#### 2.3.1 Ownership concentration

The management literature explains ownership concentration in terms of the extent of homogeneity of firm ownership [39]. Whereas ownership concentration reduces the challenges of management rights, it is also a source of firms’ financial constraints as the financial burden of the firms falls to a few majority shareholders [40]. Within CEE, the transition from a centrally planned economy to a market economy bequeathed to individuals and groups relics of former state-owned businesses [41]. Ownership of SMEs is therefore less dispersed within CEE because most SMEs are family businesses [42]. Family involvement is important in SME management in CEE. The management and control of SMEs within CEE are normally in the hands of family members, and multiple generations have been involved in the management of SMEs [43]. As a result, the decision-making power and responsibility of financing organizational activities are given to the family.

Firms’ management capabilities are considered another important determinant of firms’ innovation performance [1]. The literature on firm ownership makes clear that decision making in firms is greatly influenced by their ownership [1, 44]. Within the SME sector, ownership concentration can be associated with the influence in management decision-making [16]. In this light, they argued that SMEs’ performance is directly linked with firms’ ownership.

Chatterjee and Bhattacharjee’s [16] findings indicate that Indian SMEs’ technology performance is positively influenced by the interaction between the promoter’s ownership concentration and R&D intensity. The empirical literature on the moderating role of ownership concentration in the relationship between R&D intensity and innovation presents firms’ ownership concentrations as an important factor explaining why the relationship between R&D and innovation differs among firms. Single-owner firms convert R&D to innovation more efficiently than firms with multiple owners [45]. Deng et al.’s [45] findings are based on data from 43,728 Chinese firms between 2005 and 2006. The agency cost is minimised with concentrated ownership; thus, conflict between owners is also minimised [46]. Concentrated ownership aligns interests [47], allowing firms to focus on R&D activities more easily to improve innovation. It is important to stress that, in as much as ownership concentration may be a recipe for enhancing the relationship between R&D and innovation, empirical findings show that an excess concentration of ownership reduces the positive relationship between R&D and innovation in firms [48].

Considering the fact that R&D intensity is a reflection of a firm’s long-term strategy to develop its capabilities [49], stakeholders with long-term interests, such as owners with the largest shares, are expected to influence firms towards increased innovation performance. We focus on firms within the CEE region because, to our knowledge, there is limited empirical evidence on the relationship between ownership concentration and firm innovation. When considering previous studies’ findings, it is possible to see the relationship between ownership concentration and company performance in general. Claessens and Djankov [50] stated that firms’ more concentrated ownership triggers their performance in terms of profitability and labour productivity in the Czech Republic. In contrast, Balsmeier and Czarnitzki [51] showed that the relationship between ownership concentration and firm performance is an inverted U-shape. Meanwhile, Iwasaki and Mizobata [52] confirmed their expectation of the positive influence of firms’ ownership concentration on performance by analysing the emerging economies of CEE. Therefore, the current study takes the positive position that, in the presence of high ownership concentration, where a large portion of the firm is owned by the one or a few owners, firms will be directed towards enhanced R&D for increased innovation. We hypothesize that:

***H***_***3***_: *Ownership concentration positively moderates the relationship between R&D intensity and product innovation in CEE countries*.

#### 2.3.2 Private versus public ownership

Within the CEE region, the shift from central planning to market economy also shifted the control of businesses from public to private. Yordanova et al. [43] explained that the transition from central planning to a market economy in the CEE region led to the emergence of privately owned SMEs and family businesses. The modern thinking of business organization in the CEE is a shift towards private engagement in businesses, which means that decision-making and financing of businesses in the CEE region fall to the shoulders of the private sector. There is empirical evidence to suggest that many private firms as well as SMEs are first-generation family businesses managed by their elderly founders [53]. The public sector involvement in SMEs is minimal in CEE countries. This is in compliance with a shift in the thinking of economic management towards a private sector lead policy. Although the individual CEE countries show different levels of private involvement, the dominant position is that private ownership of business is on the rise [54, 55].

Private ownership of firms has been touted as explaining a significant portion of firm innovation. Using data from ten thousand firms across 34 developing countries, Ayyagari et al. [56] showed that more innovative firms are export-oriented privately owned firms rather than state-owned ones. Hussen and Cokgezen [57] made similar observations using World Bank survey data on Ethiopia. Specifically, the authors found that firms’ private ownership has a positive effect on process innovation but not on product innovation of firms. The findings of Gao et al. [58], who compared innovation strategies of private and public owned firms, confirm the position of Ayyagari et al. [56] and Hussen and Cokgezen [57]. Gao et al. [58] observed that private firms are more innovative than public firms because, whereas private firms are explorative in their innovative activities, public firms have been exploitative. Private ownership of firms includes the right to ownership of assets, which gives the owner control and bargaining power [59]. The incentive to strengthen investment in cost reduction in the use of assets has been proven to be motivated by private ownership as the benefits accrue to the owner. Private ownership of firms is, therefore, a source of improved investment in innovation.

Within CEE countries, there is empirical evidence to suggest that state-owned enterprises are less innovative than privately owned firms. Ullah [60] analysed the source of the performance gap between privatised and private firms within CEE and Central Asian countries. The findings show that innovation is the main determinant of firm performance; however, the performance gap between private firms and privatised firms stems from private firms within the sampled area being more innovation oriented than state-owned firms that were privatised. The author attributed this disparity to privatised firms holding onto some of the tenets of state-owned enterprises whereas private firms remain sensitive to innovation for improved performance. The organic profit motives of originally private firms have been the main driver to innovate, contrary to the less pro-innovation attitude of originally state-owned enterprises that have been privatised [60].

In addressing the question on whether private or public ownership improves innovation, a study by Ferreira et al. [61] showed that public sector firms are more inclined to exploit existing knowledge for innovation, which is contrary to the private sector, where exploration for innovation is the norm. Based on the prior literature, the following hypothesis is formulated:

***H***_***4***_: *Private domestic ownership of firms positively moderates the relationship between R&D intensity and product innovation in CEE countries*.

#### 2.3.3 Family versus non-family ownership

An important aspect of firm ownership that has emerged in the innovation literature is family ownership of firms and how it impacts firm innovation. Although the literature makes explicit that R&D intensity improves firm innovation, there are scant studies on the moderating role of family ownership in this relationship. For example, Sanchez-Famoso et al. [62] investigated the moderating effect of family ownership in the relationship between the family’s social capital and firm innovation. The authors relied on data from 172 Spanish family firms and observed that the higher the ownership of the family is, the more both family and non-family social capital enhances firm innovation. The role of family ownership in enhancing firms’ innovation performance is confirmed in related studies. Using data from Taiwanese firms, Chen et al. [63] showed that family-owned firms have more incentives to encourage innovation investment. This is confirmed in a study that assessed how family businesses contribute to innovation performance in India. Lodh et al. [64], using unbalanced panel data of 365 Bombay listed firms owned by Indian families between 2001 and 2008, revealed the significance of family ownership of firms in innovation performance. However, family ownership of businesses determines whether firms will be exploitative or explorative in their innovation. Cognitive psychologists have shown that the extent to which family owners of firms value external knowledge more than existing knowledge changes the longer a family has ownership [65]. Longer family ownership leads to emotional attachment to ideas and the challenges stemming from external knowledge not being accommodated. As a result, the firm will adopt an exploitative innovation strategy rather than an explorative innovation strategy. Whereas non-family owners of firms are more inclined towards innovation, family-owned firms become more inclined to exploitative innovation strategies over time.

In the context of the CEE region, the literature has been contentious. Contrary to the what the literature in general has posited, there is evidence that in Poland, for example, family involvement in business reduces innovation. Thus, although studies on firm ownership’s effect on firm innovation abound, studies in CEE countries are rare despite the increasing formation of innovation policies and regional innovation systems in these countries [66]. It is instructive to note that, in CEE countries, there is high public funding of innovation activities. For example, in the Czech Republic, of the 2% of the GDP that goes to support R&D, 68% is financed by the government [66]. CEE countries have deployed policy measures such as R&D subsidies and tax credits that allocate resources for R&D activities in firms. Ownership of firms is therefore important for understanding how these government supports can translate into innovation performance. Yet even with these R&D support systems in place, economies of CEE countries continue to lag behind in scientific-related economic activities, making it imperative to examine how firms’ different ownership structures that are critical in firm decision-making influence innovation activities in firms.

Considering all of the arguments presented thus far, the study postulates the following hypothesis:

***H***_*5*:_ *Family ownership positively moderates the relationship between R&D intensity and product innovation in CEE countries*.

## 3. Data and methods

### 3.1 Data source

Data for the study came from the World Bank Enterprise Survey 2019. The choice of data was consistent with earlier studies that have also used the World Bank Enterprise Survey for related studies (see [67, 68]. The World Bank Enterprise Survey gathers information and opinions about countries’ business environments. These firm-level data cover 16 areas of a firm’s business environment. Areas of focus of the survey which influenced the selection of this choice of data source included general business information, infrastructure and services, sales and supplies, management practices’ degree of competition, innovation, and capacity.

Although the World Bank Enterprise Survey provides cross-sectional data on many countries, the countries of interest for the current study were the CEE countries of the Czech Republic, Bulgaria, Latvia, Poland, Lithuania, and Slovakia. In all, 136 firms are sampled in an imbalanced manner from these CEE countries for the current study. In contrast, as evidenced by prior research performed within emerging European innovation systems [11, 17], these selected countries and the companies in them are characterised by similar features; therefore, it is common for them to be combined as one representative sample for these types of analyses. This approach allowed us to get a better picture of the entire region under study and, thus, propose implications that will be valid for the wider whole. We subsequently controlled our findings for different sectors (see Section 4.2). In this study, we sampled 30 firms from the Czech Republic, 18 from Bulgaria, 19 from Latvia, 23 from Poland, 21 from Lithuania, and 25 from Slovakia. The sampled firms were drawn from five sectors of the economy: manufacturing, wholesale, construction, hotel and restaurant, and the services sectors.

Table 1 presents the sectors of the economy from which the sample for the study was drawn. As indicated, 77 firms in the sample, representing 56.6%, were drawn from the manufacturing sector, followed by 18 wholesale sector firms (13.2%), 16 construction sector firms (11.8%), 13 service sector firms (9.6%), and 12 hotel and restaurant sector firms (8.8%). The average number of employees at the sample firms was 91.29. The average number of individuals in the sectors ranged from the manufacturing sector (126.8) to the wholesale sector (65.33). As the World Bank Enterprise Survey on CEE countries is missing some data on the different sectors, the study sampled firms based on completeness of data and fewer than 250 employees. The limit in the number of employees was used to capture SME firms consistent with the European Commission’s definition as cited in Mateev and Anastasov [69].

**Table 1:** Sampled sectors and sample size.

Sectors	Sample	Percentage	Average Number of Employees
Manufacturing	77	56.6	126.84
Wholesale	18	13.2	65.33
Construction	16	11.8	72.12
Hotel and Restaurant	12	8.8	107.46
Service	13	9.6	89.70
Total sample	136	100	91.29

Source: Authors’ Construction

### 3.2 Variable descriptions and measures

Nine variables were used in this study. Based on the theoretical and empirical literature, examining the moderating effect of different ownership structures in the relationship between R&D intensity and product innovation involved the use of variables and the measures outlined in Table 2. In measuring product innovation, we were inspired by Teng and Jingtao [10], who measured product innovation based on the ratio of new product sales to total sales. The intuition is that product innovation is captured as the value of sales earned from the sale of new innovative products per the firm’s total sales. The study adopted this measure in line with the available data.

**Table 2:** Variables and measures.

Variables	Measures	References
**Dependent Variables**		
Product Innovation	Ratio of new product sales to total sales	Liu and White [47], Faber and Hesen [70], Carvalho et al. [71], Teng and Jingtao [10]
**Independent Variables**		
R&D intensity	R&D expenditure/total sales	Francis and Smith [72], Ortega-Argiles et al. [73], Kor [74], Sciascia et al. [75], Teng and Jingtao [10]
Capacity utilization	Output as a percentage of maximum possible output	Wang and Li [76]
**Moderating variables**		
Family control	% of firm owned by family	Islam et al. [77]
Private ownership	Ratio of private-owned assets to total assets if a firm is private-owned	Islam et al. [77]
Ownership concentration	% of firm owned by the largest owner(s)	Deb and Chaturvedula [78], Chatterjee and Bhattachajee [16]
**Control variable**		
Research Capacity	Internal R&D expenditure	Zahra and George [79], Todorova and Durisin [80], Zou et al. [81]
Firm size	Log of total employees	Herrera and Sánchez-González [82], Teng and Jingtao [10]
Process innovation	1 if firm introduced new production process, otherwise = 0	Kahn, Sithole and Buchana [83]

The World Bank Enterprise Survey provides data on firms’ sale of new products and total sales, making this measure ideal. Several studies have used the same measure to capture product innovation, including Faber and Hesen [70] and Carvalho et al. [71]. Family ownership is another important variable that moderates the relationship between R&D intensity and product innovation in our study. Motivated by Islam et al. [77], who measured family ownership as the ultimate ownership or ultimate equity in a listed firm directly or indirectly by a family, we applied this concept to our data.

Consistent with Islam et al.’s [77] measure of family ownership, the World Bank Enterprise Survey reports on the percentage of a firm owned by the family, which the current study adopted as a measure of family ownership. Following logically from Islam et al. [77], ownership is about the asset size that is owned; it is by extension acceptable to argue that private ownership is measured by the proportion of a firm’s total assets owned by the private sector. This forms the basis of our measure of private ownership given the data available. The World Bank Enterprise Survey asks a specific question to solicit the percentage of firm assets owned by private individuals, companies, or organizations. The World Bank Enterprise Survey data solicit information on the percentage of a firm’s assets owned by the largest owner or owners, which is consistent with the thinking of Chatterjee and Bhattachajee [16] that the promoter’s equity measures the ownership concentration of the firm. The R&D intensity measure adopted in this study was inspired by the work of Sciascia et al. [75] and Teng and Jingtao [10], who measured R&D intensity as a proportion of firms’ R&D expenditures to total sales revenue. Although there are a plethora of other measures, the concepts are the same as the measures adopted. The choice of measures was motivated by the data available. In measuring firms’ capacity utilisation, Wang and Li [76] inspired the choice of measure. They measured capacity utilisation as the ratio of actual output to potential output if all resources are fully applied.

Table 3 presents the descriptive statistics of the data used for the study. As shown, product innovation has a mean of 79.38 and a standard deviation of 29.9. Thus, 79% of firms’ sales are a result of innovation. Innovation ranges from as low as 1% to 100%. The data on innovation included 136 observations. Process innovation was measured as a dummy variable.

**Table 3:** Descriptive statistics.

Variables	Mean	Standard deviation	Median	Min	Max	Observations
Product Innovation	79.38	29.90	90	1	100	136
Process innovation	0.911	0.286	1	0	1	136
Capacity utilization	70.71	32.84	80	1	100	136
Firm size	95.97	132.32	37	1	600	136
Research capacity	513227.32	1371445.20	35000	1	100000000	136
R&D intensity	0.02	0.04	0.002	4.46429E-10	0.2	136
Ownership concentration	81.15	26.16	100	17	100	136
Private domestic ownership	87.28	31.43	100	0	100	136
Family ownership	66.13	41.41	92.5	0	100	136

The three control variables used in this study have been empirically proven to influence innovation and R&D outputs. Research capacity measured by internal R&D expenditure determines the extent of firms’ innovation activities and the extent to which firms can assimilate external knowledge [79–81]. Firm size as a control variable in the innovation model is important because the size of the firm determines a lot about the firm, including financial capacity and consequently the firm’s ability to invest in R&D activities. Including process innovation as a control variable in a product innovation model is logical as it includes all the new ideas involved in an organization’s activities that lead to producing its outputs. An important component of the product innovation model is process innovation [85]. It is therefore important to account for the contribution of process innovation in the product innovation model. Although the use of dummy variables as a measure of process innovation has the shortcoming of not being able to capture the extent of innovation intensity, the nature of the data we had left no room to use other measures of process innovation than a dummy variable. Indeed, a large body of innovation literature has measured process innovation through dummy variables [83]. These control variables are introduced to reduce biases in the regression model. The mean of 0.911 indicates that 91.1% of the firms experienced innovation in their processes. The average capacity utilisation is 70.71%, with a standard deviation of 32.84%. The average firm size of the firms sampled in the study was 95.97, with a standard deviation of 132.32. The average R&D intensity of the sampled firms was 2% of sales, with a standard deviation of 4% of sales. The maximum R&D intensity was 20% of sales while the minimum R&D intensity was almost 0.0%. Ownership, measured by ownership concentration, had a mean of 81.15%, indicating that on average 81% of the sampled firms were owned by the largest owner or owners. In addition, 87% of the sampled firms were owned by private domestic individuals, firms, or organizations while 66% were owned by families.

Table 4 presents the correlation analysis of the variables that enter the regression model. The correlation between the variables was low enough (< 0.7 absolute terms) to suggest the absence of multicollinearity in the regression model.

**Table 4:** Correlation analysis.

	*Prod_inno*	*RD_int*	*Proc_inn*	*Cap_ut*	*Firm_size*	*Res_cap*	*Own_con*	*Priv_dom*	*Fam_on*
*Prod_inno*	*1*.*0000*								
*RD_int*	*0*.*1536*	*1*.*0000*							
*Proc_inn*	*-0*.*3521*	*-0*.*0878*	*1*.*0000*						
*Cap_ut*	*0*.*3451*	*0*.*1719*	*-0*.*0575*	*1*.*0000*					
*Firm_size*	*0*.*2597*	*-0*.*1235*	*-0*.*0650*	*0*.*2482*	*1*.*0000*				
*Res_cap*	*0*.*0408*	*0*.*4282*	*0*.*0153*	*0*.*0572*	*0*.*0475*	*1*.*0000*			
*Own_con*	*0*.*0182*	*-0*.*0078*	*0*.*0289*	*0*.*0289*	*0*.*0592*	*-0*.*1522*	*1*.*0000*		
*Priv_dom*	*0*.*2729*	*0*.*1858*	*-0*.*0178*	*0*.*2181*	*-0*.*3415*	*-0*.*0072*	*-0*.*1184*	*1*.*0000*	
*Fam_own*	*-0*.*0785*	*-0*.*1864*	*0*.*2505*	*0*.*0827*	*-0*.*1306*	*-0*.*1015*	*0*.*3684*	*0*.*0190*	*1*.*0000*

### 3.3 Research model

This study employed an ordinary least squares (OLS) regression as the estimation approach. Prior empirical studies have suggested a link between firms’ R&D intensity and product innovation [26, 84]. The OLS regression estimation approach was adopted to estimate the relationship between firms’ product innovation and R&D intensity as well as the moderation effect of ownership structure. All variables were a logarithmic transformation with the exception of process innovation. The regression model tested the effect of R&D intensity on product innovation, as shown in Eq 1.

$$Inn=\beta_{0}+\beta_{1}{proc}_{i}+\beta_{2}CapUt_{i}+{\beta_{3}{CapUt_{i}}^{2}+\beta_{4}{Fsize}_{i}+\beta }_{5}RsCap_{i}+\beta_{6}RDint_{i}+\beta_{7}Ownership+e_{i}$$ (1)

where *Inn* is product innovation, *proc* is process innovation, *CapUt* is capacity utilisation, *Fsize* is firm size, *RsCap* is research capacity, *RDint* is R&D intensity, *β* is the coefficient, *e* is the error term which is homoscedastic, and *i* is the different firms sampled for the study.

$$Inn=\beta_{0}+\beta_{1}{proc}_{i}+\beta_{2}CapUt_{i}+{\beta_{3}{CapUt_{i}}^{2}+\beta_{4}{Fsize}_{i}+\beta }_{5}RsCap_{i}+\beta_{6}RDint_{i}+\beta_{7}Ownership+\beta_{8}\;RDint*Ownership+e_{i}$$ (2)

Eq 2 tests the moderating effect of ownership structure in the relationship between R&D intensity and ownership structure represented by the interaction term ${(\beta }_{6}RDint_{i}*Ownership)$. Ownership is represented by the three moderating variables of ownership concentration, private domestic ownership, and family ownership. Thus, the study presents four regression models: a base model that examines the relationship between R&D intensity and capacity utilisation on one hand and product innovation on the other hand and three other models that capture Eq 2, where the moderating role of firm ownership, represented by ownership concentration, private domestic ownership, and family ownership, is investigated.

### 3.4 Diagnostic tests

One of the challenges of cross-sectional studies is heteroscedasticity in the error terms [85]. In this study, we conducted a heteroskedasticity test to ensure that the model was free of heteroskedasticity in order to produce accurate hypothesis testing. Logarithmic transformation of the data was employed to reduce outliers to deal with heteroscedasticity. Autocorrelation is ordinarily a phenomenon of time-series data rather than cross-sectional data. However, taking a cue from Dubin [86], who argued that an entity’s location can affect behaviour, it was imperative to assume that data for this study, which came from different countries and firms, predisposed the regression results to spatial autocorrelation. Inspired by King and Evans [87] and ensuring that the data retained its natural ordering, Durbin Watson statistics were used to test for the presence of spatial autocorrelation. A subsample analysis was also conducted to assess the consistency of the results between the two samples analysed. In this case, the manufacturing sector was sampled because it was the largest sample (56%) as well as the services sector sample to cater to the remaining sample. A model is considered good when one segment of the sample is unable to sway the results.

## 4. Results

### 4.1 Experimental results

**Fig 1:**
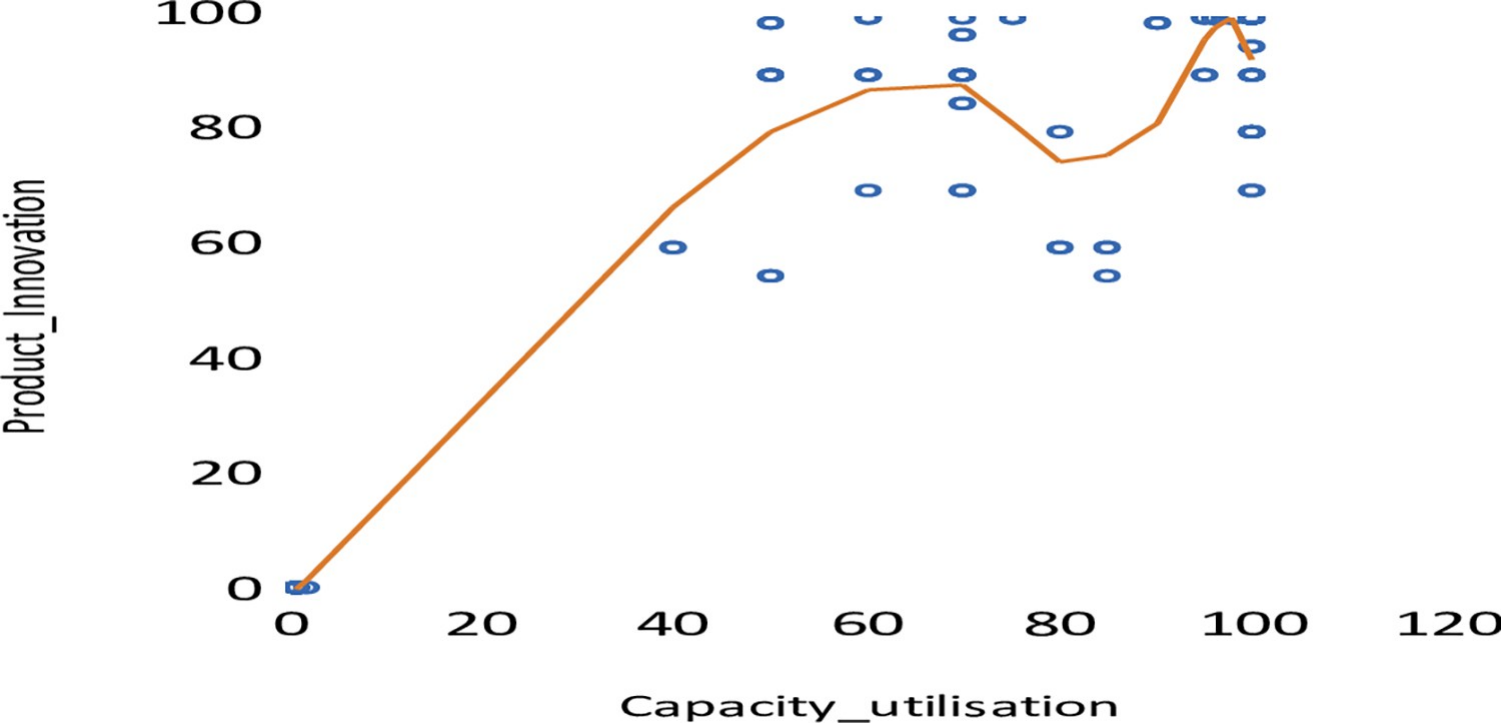
Scatter plot on product innovation and capacity utilisation.

This section of the study presents the results of the data analysis, including the full sample analysis of all data based on the sampled countries. The relationship between product innovation and capacity utilisation, as shown in Fig 1, is not linear but rather curvilinear. This indicates that fitting a linear regression will not be ideal. Therefore, the study fit a quadratic relationship to better capture the relationship between product innovation and firms’ capacity utilisation.

The results of the data analysis presented in Table 5 show four regression models. Based on the diagnostic tests, all four regression models have error terms that are homoscedastic. Savin and White [88] showed that the lower bounds of 0.0010 and 0.0002 and upper bounds of 3.9990 and 3.9998 for samples 100 and 200, respectively, indicate the absence of autocorrelation. The results from the current data analysis, with a sample size of 136, indicate the absence of autocorrelation in all four models.

**Table 5:** Regression results of full sample.

Log of Product Innovation	Model 1	Model 2	Model 3	Model 4
Independent Variables	Coefficient	Coefficient	Coefficient	Coefficient
C	-0.9744252 (2.054145)	-1.003333 (2.072084)	-6.85321 (15.61948)	-0.9072658 (2.066403)
Process innovation	-0.2409665*** (0.001342)	-0.2399163*** (0.0702252)	-0.2364171*** (0.0711336)	-0.2390551*** (0.0699977)
Capacity Utilization	0.2472361*** (0.001342)	0.2641120*** (0.022012)	0.086465*** (0.0342455)	0.006751*** (0.066243)
Capacity Utilisation^2	0.3859647*** (0.0356397)	0.3840332*** (0.0364408)	0.3817501*** (0.0375776)	0.3873*** (0.0358658)
Firm size	0.1061692*** (0.0366394)	0.1078909*** (0.0373296)	0.1084629** (0.0373991)	-0.01043538*** (0.0369152)
Research Capacity	-0.0408234** (0.0802421)	-0.0476146 *** (0.0192549)	-0.0487032*** (0.0197081)	-0.0467303*** (0.090405)
Ownership concentration	0.0408234* (0.0802421)	0.0060973** (0.1702326)	0.0560274** (0.0902038)	0.0959375*** (0.2209785)
Private Domestic Ownership	0.664195* (0.0432692)	0.6471634** (0.4432858)	1.974833** (3.479475)	0.703466*** (0.4426033)
Family Ownership	-0.0369469 (0.0432692)	-0.0510819 (0.0627522)	-0.041917 (0.0455093)	-0.2367414 (0.3037022)
R&D Intensity	0.0858952*** [0.050652] (0.0233148)	0.0642411*** (0.0730174)	1.010017*** (2.886186)	0.1285535*** (0.0683177)
R&D intensity X ownership concentration		0.0057717*** [0.0726900] (0.0184272)		
R&D intensity X Private domestic ownership			0.2389016*** [3.801619523] (0.6291487)	
R&D intensity X family ownership				-0.0108187 (0.0162758)
R-squared	0.9745	0.9736	0.9720	0.9747
Adjusted R-squared	0.9711	0.9706	0.9706	0.9708
F-statistics	282.11	246.95	247.15	248.44
Prob(F-statistics)	0.0000	0.0000	0.0000	0.0000
Prob(Homoscedasticity)	0.2271	0.3362	0.3451	0.0000
Durbin Watson	1.9331	1.9645	1.9834	1.9764

Model 4, which tests Note

***p<0.001

**p<0.01

*p<0.1. standard errors in curly brackets, standardized coefficients in square brackets. Coefficients are elasticities except for process innovation.

*Model 1* tests Hypothesis 1, which stated that R&D intensity has a significant effect on product innovation of CEE firms. Evidence from the analysis supports this hypothesis. The results show that firms’ R&D intensity positively and significantly influences firm innovation performance. Specifically, a one-percentage point change in R&D intensity translates into a 0.09-percentage point change in firms’ product innovation in the same direction. In testing Hypothesis 2, which postulated that capacity utilisation significantly affects product innovation in CEE countries, the results of the scatter plot (see Fig 1) revealed that the relationship is curvilinear, not linear. The study then conducted a second-degree polynomial regression or quadratic model. The results (see Table 5) show that the linear term is uniformly positive and significant at 1% while the quadratic term is also uniformly negative and significant at 1%. The relationship between a firm’s capacity utilisation and product innovation is therefore an inverted U-shape, suggesting that after the highest point of product innovation is reached, it begins to fall as capacity utilisation increases. Thus, the hypothesis that the relationship between capacity utilisation and product innovation is an inverted U-shape is confirmed. The relationship between firm size and firm innovation is positive whereas the relationship is negative in the case of firms’ research capacity.

In *Model 2*, the study tests Hypothesis 3 that ownership concentration positively moderates the relationship between R&D intensity and firm innovation. The data analysis results confirm this hypothesis. Thus, relative to firms with lower ownership concentration, in situations where the firm’s ownership is concentrated, the findings indicate that a change in R&D intensity leads to a greater change in innovation in the same direction. This is evident by comparing the standardized coefficients. The standardized coefficient of R&D intensity in *Model 1* is lower than that of the interaction between R&D intensity and ownership concentration in *Model 2*.

*Model 3* tests Hypothesis 4, which states that private domestic ownership of firms positively moderates the relationship between R&D intensity and firm product innovation. The analysis results support the hypothesis. Product innovation in private domestic firms is more responsive to changes in R&D intensity in the same direction. Comparing the standardized coefficients in *Model 3* to *Model 1*, it becomes obvious that the interaction between R&D intensity and private domestic ownership has a greater effect on product innovation than R&D intensity in *Model 1*.

Hypothesis 5 on the moderating role of family ownership in the relationship between R&D intensity and product innovation, was not supported based on the outcome of the data analysis. We therefore must reject Hypothesis 5.

### 4.2 Control tests

To deal with the problem of heterogeneity in our dataset and to understand its influence on our results, a control analysis was conducted using sub-samples from our data set. We split the data into two: manufacturing sector data (56.6% of our sample) and other sectors (43.4%). A large portion of our sample came from the SMEs in the manufacturing sector whereas the remainder was distributed among SMEs operating in the services sector.

Table 6 presents the results on SMEs operating in the manufacturing sector; Table 7 presents the findings on the four service sector SMEs. From Table 6, the moderating role of ownership concentration, private domestic ownership, and family ownership in the relationship between R&D intensity and product innovation was analysed. Table 6 also presents the results on the role of firms’ capacity utilisation in product innovation for SMEs in the manufacturing sector. Consistent with the full sample data presented in Table 5, the subsample data results show the importance of firms’ capacity utilisation in product innovation. An inverted U-shaped relationship existed between firms’ capacity utilisation and innovation performance among SMEs in the manufacturing sector. The subsample data analysis also confirmed the significance of R&D intensity in affecting product innovation as well as the moderating role of ownership concentration in the relationship between R&D intensity and product innovation, thereby suggesting an ownership concentration additionality effect. Judging from the size of the standardized coefficients of the interaction between R&D intensity and ownership concentration in *Model 2* and R&D intensity in *Model 1*, the standardized coefficient was higher for the interaction terms in *Model 2* than for R&D intensity in *Model 1*. The same level of consistency between the full sample and the subsample results was observed in the case of *Model 3* and *Model 4*.

**Table 6:** Regression results of the manufacturing sector.

Log of Product Innovation	Model 1	Model 2	Model 3	Model 4
Independent Variables	Coefficient	Coefficient	Coefficient	Coefficient
C	-0.883423 (0.232321)	-0.026513 (0.207162)	-0.653111 (0.564732)	-0.876432 (0.987564)
Process innovation	-0.645432*** (0.242428)	-0.098767*** (0.009876)	-0.645231*** (0.099878)	-0.534521*** (0.120980)
Capacity Utilisation	0.345242*** (0.564631)	0.334112*** (0.00123)	0.0453625*** (0.876453)	0.001234*** (0.112435)
Capacity Utilisation^2	-0.232212*** (0.123221)	-0.242552*** (0.022343)	-0.231211*** (0.102322)	-0.243110*** (0.023342)
Firm size	0.354211** (0.023211)	0.334212*** (0.033120)	0.3345241 (0.033121)	0.323221 (0.02212)
Research Capacity	-0.033232*** (0.002321)	-0.023121** (0.023212)	-0.032121*** (0.132121)	-0.023122*** (0.034211)
Ownership concentration	0.213212 (0.021333)	0.0.21332 (0.035421)	0.011231 (0.201232)	0.423121 (0.002321)
Private Domestic Ownership	0.415232** (0.034512)	0.344321** (0.021322)	0.422314** (0.001231)	0.456342*** (0.022321)
Family Ownership	-0.434212 (0.065411)	-0.632120 (0.005432)	-0.552113 (0.027821)	-0.422112 (0.124511)
R&D Intensity	0.072132*** [0.0531242] (0.003210)	0.044291*** (0.002134)	0.432431*** (0.017720)	0.113234*** (0.0765670)
R&D intensity X ownership concentration		0.066231*** [0.0754321] (0.029876)		
R&D intensity X Private domestic ownership			0.545321*** [0.978665] (0.300187)	
R&D intensity X family ownership				-0.650921 (0.413210)
R-squared	0.8654	0.8221	0.8342	0.9747
Adjusted R-squared	0.8554	0.8001	0.9706	0.9708
F-statistics	432.10	412.71	233.21	248.44
Prob(F-statistics)	0.0000	0.0000	0.0000	0.0000
Prob(Homoscedasticity)	0.5333	0.3897	0.3661	0.0000
Durbin Watson	2.001	2.110	1.974	1.932

***p<0.001

**p<0.01

*p<0.1.

standard errors in curly brackets, standardized coefficients in square brackets. Coefficients are elasticities except for process innovation.

**Table 7:** Regression results of wholesale, construction, hotel and restaurant, and service subsectors.

Log of Product Innovation	Model 1	Model 2	Model 3	Model 4
Independent Variables	Coefficient	Coefficient	Coefficient	Coefficient
C	-0.0675342 (2.623111)	-0.0856341 (0.142321)	-0.0023102 (0.303423)	-0.0011210* (0.231100)
Process innovation	-0.2312442*** (0.1221234)	-0.2782110*** (0.0891232)	-1.143001*** (0.1203421)	-0.221324*** (0.0012432)
Capacity Utilisation	0.2342211*** (0.311234)	0.5342113*** (0.034531)	0.1342412*** (0.102342)	0.2312112*** (0.0144210)
Capacity Utilisation^2	-0.2987542*** (0.1438761)	0.4352300*** (0.609831)	0.4510881*** (0.541211)	0.66323410*** (0.0987221)
Firm size	0.02431213** (0.1543420)	0.2332011** (0.0034524)	0.1142310* (0.1239871)	0.0223121** (0.0231091)
Research Capacity	-0.2341310*** (0.1234213)	-0.001232*** (0.103201)	-0.1134564*** (0.121098)	-0.0023421*** (0.0223401)
Ownership concentration	0.09874623*** (0.0234231)	0.0143221*** (0.3425130)	0.0321123*** (0.012324)	0.1393110*** (0.0871126)
Private Domestic Ownership	0.0986453*** (0.042551)	0.0231213*** (0.3423211)	0.2310021*** (0.1234231)	0.0652144* (0.2001245)
Family Ownership	-0.2341321** (0.1098342)	-0.0123121* (0.0564633)	0.6682210 (0.0023451)	0.4536102 (0.1325340)
R&D Intensity	0.01254351*** [1.93959E-05] (0.013241)	0.00124352*** (0.003219)	0.0987341*** (0.116223)	0.0078611*** (0.0234123)
R&D intensity X ownership concentration		0.02346621*** [0.031371] (0.034120)		
R&D intensity X Private domestic ownership			0.2340941*** [0.4163683] (0.012334)	
R&D intensity X family ownership				0.022311 (0.1023441)
R-squared	0.79324	0.79812	0.81200	0.77341
Adjusted R-squared	0.78222	0.78733	0.80211	0.75990
F-statistics	365.30	371.55	359.21	366.46
Prob(F-statistics)	0.0000	0.0000	0.0000	0.0000
Prob(Homoscedasticity)	0.331	0.272	0.301	0.234
Durbin Watson	2.022	2.076	2.001	1.988

***p<0.001

**p<0.01

*p<0.1. standard errors in curly brackets, standardized coefficients in square brackets. Coefficients are elasticities except for process innovation.

Table 7 presents the results of the SMEs operating in the four service sectors and shows that, just like the case of the full sample in Table 5 and the manufacturing subsample in Table 6, the relationship between capacity utilisation and product innovation is an inverted U-shape. In agreement with the full sample and the manufacturing subsample, the service sector subsample in Table 7 also shows the moderating effect of ownership concentration and private domestic ownership in the relationship between R&D intensity and product innovation. Consistent with the full sample and manufacturing subsample, the service sector subsample also showed no moderating effect of family ownership.

Although there is a general consistency between the full sample and subsample analysis results, we observed some expected differences between the two analyses in the manufacturing and service sectors. The full sample analysis in Table 5 shows a higher coefficient of determination (R-squared) than the subsample analyses in Tables 6 and 7. In both the full and subsample analyses, the results are robust to heteroskedasticity, autocorrelation, and multicollinearity.

## 5. Discussion

This study confirmed our expectations and hypotheses that R&D intensity and capacity utilisation of firms significantly affect product innovation within the analysed CEE region, including the Czech Republic, Bulgaria, Latvia, Poland, Lithuania, and Slovakia. Both findings support the results of prior literature. R&D intensity is considered a key driver of innovation performance [24]. The literature suggests that firms’ R&D intensity builds their capacity to absorb external knowledge and carry out innovation [25], which explains why increasing R&D intensity improves product innovation. Firms that have invested a substantial amount of resources into R&D are generally expected to improve their innovation output. The findings of this study suggest that, on average, firms in CEE countries spend 2% of their incomes on R&D. This meagre spending on R&D may possibly explain why the innovation performance of firms in CEE is low relative to other countries in the European Union [89].

Likewise, firms’ capacity utilisation also determines product innovation. However, the relationship between capacity utilisation and innovation, in agreement with Nohria and Gulati [32], shifts from positive to negative after realising an optimum level of capacity utilisation. Fevolden and Gronning [31] argued that capacity utilisation could be inimical to product innovation in some instances. The source of this adverse effect on innovation is the capacity utilisation’s draining of a firm’s resources and innovation. The quadratic relationship is a confirmation of the work of Acar et al. [90], who suggested that extreme levels of capacity utilisation are not good for innovation performance. A large body of knowledge in the extant literature has suggested that reduced capacity utilisation is a good recipe for innovation performance. If we instead consider resources for innovation, it is not unthinkable to assume that these are the same resources needed for other organizational activities. Extreme capacity utilisation could mean siphoning resources from innovation activities. This is intuitive in the sense that risk-averse management would find innovation activities less lucrative in difficult times and only engage in innovation activities when there are resources to spare (i.e., slack resources).

Moreover, as Acar et al. [90] argued, slack resources make space for innovation. At lower levels of capacity utilisation, there are large expanses of slack resources that can be channelled into innovation activities. These slack resources cause a rise in innovation at lower levels of capacity utilisation. At higher levels of capacity utilisation, firms are drained of slack resources, and resources for innovation are reduced, resulting in reduced innovation. Lean production as a means of minimising waste has been critical for firms to save resources for innovation while ensuring capacity utilisation [31]. In the specific context of CEE countries, the emerging economies are not as robust; consequently, the firms are risk averse. R&D expenses in these firms will be very much affected by economic shocks. The uncertainties of innovation outcomes will have a strong bearing on how many resources are committed to innovation activities. Slack resources are therefore expected to form a large component of innovation activities, without which innovation declines.

The study findings on the moderating effect of firm’s ownership in the relationship between R&D intensity and product innovation are mixed for the CEE territory. The results confirm that firms’ ownership concentration significantly and positively affects the relationship between R&D intensity and product innovation of the selected CEE firms. In the same vein, the study confirms that private domestic ownership of CEE firms also significantly and positively affects the relationship between firms’ R&D intensity and product innovation. However, firms’ family ownership did not significantly moderate the relationship between R&D intensity and product innovation within the analysed emerging innovation systems of CEE firms and countries.

These findings support Chatterjee and Bhattacharjee’s [16] results. These authors argued that firms’ ownership concentration in CEE countries improves the effect of R&D intensity on innovation performance. Contrary to the findings of the current study, Su et al. [23] suggested that ownership concentration is not critical to innovation performance. The authors argued that, when ownership is concentrated, the burden of R&D investment rests with a smaller group of investors who may not be willing to bear the risk of innovation. It is important in the context of the CEE region to recognise the role of government support [5]. Ownership concentration positively moderates the relationship between a firm’s R&D intensity and innovation performance within CEE, despite Su et al.’s [23] risk argument, because the government’s strong support for firms’ innovation helps deal with any risk associated with innovation in firms. Within the CEE territory, firms benefit from R&D support, which takes away part of the risk to which Su et al. [23] alluded, thereby making it easier and more enticing for even risk-averse firm owners to invest in R&D and increase the resource pull for firms to tap into for innovation activities.

Intuitively, decisions about innovation will not be easy to make and will have long-term implications. Decision-making is more likely to be easier when ownership is concentrated than when it is less concentrated. Jiang and Peng [46] showed that agency cost in terms of conflict between owners is minimised with concentrated ownership. The long-term gains of R&D expenditures today are more likely to resonate with concentrated ownership because of the small number of people involved and the likelihood of easily rallying others to understand the gains of R&D expenditures relative to convincing larger groups of people in the case of dispersed ownership. The literature on ownership presents two sides of ownership concentration, both of which have important bearings on the findings of the study. On one hand, ownership concentration is a financial constraint to innovation; on the other hand, decision-making rests with a small majority shareholders when ownership is concentrated [16]. As previously argued, within the CEE region, government support helps deal with the issues of financial burden. Although the burden of financing innovation rests with a smaller group of owners in situations of ownership concentration, we argue that government support by way of R&D has enabled CEE firms to deal with the financial burden of innovation activities. Another side of the argument is that swift decision-making could be capital for R&D activity. Decisions about investing in innovation activities are strategic and require strong resource commitment. A decision of this nature will not be easy to make. Ownership concentration in this case will be an asset as it will foster quick decision-making, unlike dispersed ownership.

The moderating effect of private domestic ownership in the relationship between R&D intensity and product innovation, as revealed in this study, concurs with the findings of Ayyagari et al. [56], Gao et al. [58], and Hussen and Cokgezen [57], who found that firms’ private ownership positively relates to innovation performance. These findings corroborate the positive moderating effect of firms’ private ownership in the relationship between R&D intensity and firm innovation. This may suggest that firms’ profit motives drive them to invest resources in R&D to improve innovation. Ullah [60] demonstrated that private firms’ desire to make a profit drives them to engage in innovation. In the face of risk associated with R&D investment and inadequate funds for R&D, firms will require motivation to commit to R&D investment. The findings of this study point to the organic profit motive of private firms [60] and, in the CEE context, government support [5]. The findings further indicate how CEE government R&D support for firms contributes to reducing R&D investment risk by providing firms with resources to contribute to innovation. The positive moderation effect of the private domestic ownership in the relationship between R&D intensity and innovation performance could be explained partly by the explorative nature of private firms. Gao et al. [58] showed that private firms are more explorative than exploitative in their R&D activities, meaning that—rather than looking inward—private ownership inclines firms to open innovation where R&D resources are externally sourced in combination with internal R&D resources. Such an orientation opens doors to a higher pool of efficient R&D resources to shore up innovation. Indeed, Ferreira et al. [61] found that explorative innovation is more strongly motivated by private ownership than by public ownership.

The findings of the current study that family ownership does not moderate the relationship between R&D intensity and product innovation within the CEE region contradicts previous findings. For example, Chen et al. [91] and Sanchez-Famoso et al. [62] found that family ownership catalyses firms’ innovation. The CEE context may be important in explaining why family ownership does not significantly moderate the relationship between R&D intensity and product innovation. SMEs in CEE are managed by first-generation owners who are older [53] and have lost the momentum needed for innovation. The results of this study may raise questions about how knowledgeable these family owners are. Steinerowska-Streb and Wziątek-Staśko [92] concluded that family ownership of businesses in Poland, for example, could translate into strong innovation outcomes only when the owners are knowledgeable enough to engage in innovation.

## 6. Conclusions

### 6.1 Summary and contributions

Firms’ R&D intensity is an important determinant of their product innovation. The relationship between R&D intensity and innovation performance has received attention in the literature, especially in the Western and Asian economies, which are characterised by advanced technology industries. This paper empirically assessed the moderating role of different ownership structures in the relationship between firms’ R&D intensity and product innovation as well as the effect of capacity utilisation in their innovation performance. Previous studies have generally investigated the role of R&D intensity or the effect of firm ownership characteristics on firms’ innovation performance. With regard to capacity utilisation and its relationship to firms’ innovation performance, prior literature has provided scant information, assuming a linear relationship between firms’ capacity utilisation and innovation performance. The current study analysed the relationship between capacity utilisation and innovation performance while bearing in mind that increasing firms’ capacity can lead to diminishing returns, thereby requiring an estimation of the relationship using polynomial regression.

The unique results of this study set it apart from prior literature findings: (i) there is an inverted U-shaped relationship between firms’ capacity utilisation and innovation performance and (ii) firms’ capacity utilisation leads to diminishing returns as more and more capacity is used to improve firm innovation. Rather than assuming that firms’ R&D decisions are made without the influencing effect of the ownership structure, we took a practical approach that the firm’s ownership structure influences its R&D investment to understand how it affects innovation performance. The study concludes that ownership concentration and private domestic ownership of firms are complementary with firms’ R&D intensity in enhancing innovation performance within the CEE region.

### 6.2 Managerial and policy implications

In terms of policy implications, based on the findings of this study, policymakers and managers in CEE economies should design incentive policies to support firms’ R&D and innovation efforts. R&D activities are expensive and come with high risk, which is one reason why firms may not be willing to engage in such activities. Government support for R&D can provide insurance against the risk of investing in R&D and support to increase R&D activities. It is not enough to simply provide funds for R&D activities; the support must be comprehensive to the core. In line with previous research [93], policies towards improving innovation performance should cover a broad range of policy actions that encapsulate improving the governance structure, policy instrument, and policy process as well as building institutional capacity. The necessary needed legislation must be put into place to assign specific institutions the task of overseeing the implementation of innovation policies. One area that has proven to be challenging when it comes to innovation within SMEs is funding due to the firms’ small size. Policy instruments for improving innovation within the SME sector must be geared towards improving funding for innovation. Other areas the instrument could target include technology parks to generate knowledge that could benefit those SMEs not in a position to finance their own R&D.

Given the huge knowledge and technical skills that innovation activities demand, SMEs with their limited financial resources are generally not in a position to single-handedly improve innovation performance. Collaboration is therefore an important source of learning for these SMEs. Government funding for SME R&D could be targeted at specific areas, such building firms’ R&D capabilities. The cost of capacity building for R&D has always be a concern for governments. The most effective ways of dealing with these costs is to centralise R&D capacity building [94] rather than having each SME have its own capacity-building system.

In addition, firms are encouraged to avoid operating at extreme capacities and instead operate between extremes, which is considered the optimal level of capacity utilisation for innovation performance. The search for an optimum level of capacity is not an easy task. However, an important guiding principle is to ensure that capacity utilisation does not reduce resources available for innovation activities. As suggested by Fevolden [20], one approach of combining capacity utilisation and increased innovation performance is to adopt lean production and flexible production. This approach frees resources for activities such as R&D without compromising firms’ ability to utilise resources fully. The principle of lean production fits SMEs’ bid to improve innovation, especially because of the resource constraints SMEs face due to their small size. SMEs generally do not make gains from economies of scale; therefore, the efficient use of resources is imperative for innovation performance. Lean production can enable SMEs to maximise productivity while minimising waste [95]. These cost savings from reduced waste provide extra resources to invest in innovation activities.

Family ownership did not moderate the relationship between R&D intensity and product innovation in CEE countries, but we did propose several implications for family firms and their owners and managers. As a number of family firms in the CEE region are managed by first-generation owners [53], it is necessary to understand their fundamental characteristics and concerns, which can act as barriers to the innovation process. Such barriers can include, for example, risk aversion and striving more for the continuation of the family dynasty and stable growth than for the uncertainty associated with the innovation process. Therefore, family firms in CEE territories should understand the benefits associated with innovation while simultaneously trying to be open to the outside world, such as by hiring non-family managers who can support firms’ innovative potential and absorptive capacity. These steps could also increase CEE family firms’ internal and external social capital [96, 97], which is generally less developed in CEE countries [11]. Moreover, we recommend that these firms focus on educating next-generation leaders, although such steps must be carried out quickly enough for CEE family firms to keep up with current trends and competition [98]. Finally, in general, greater openness is recommended for family firms, which often try to protect their socio-emotional wealth and do not see the potential benefits of openness and cooperation, such as the flow of new external knowledge, resources, and experience [99].

### 6.3 Limitations and future research

This study is limited to investigating the relationship between firms’ capacity utilisation in the CEE region and their innovation performance. Attempts to generalise the findings of the study should therefore remain within the bounds of the study. Future studies should use data and estimation techniques that will make it possible to determine the exact capacity utilisation level that is optimal for innovation performance within the CEE region. The farthest the current study could go was to determine that the optimal capacity utilisation level for innovation falls between the two extremes of capacity utilisation. The study used a rather small sample size, and the structure of the data compelled the study to adopt a cross-sectional analysis, which is less informative relative to a panel data structure, which would allow the study to follow firms over time and across space.

An important feature of small firms in the CEE region is that they are privately owned and managed by older founders [53]. One area of interest that future researchers should consider is how this feature affects innovation in the CEE region, which combines two opposite features in terms of innovation. Although older owners may have lost momentum for innovation, private firms are fertile ground for innovation. Future studies should assess which of these opposing factors determines the outcome of innovation in SMEs. Moreover, in line with the current study’s focus on the CEE region, we also recommend the following avenues for future research: effects of digitization on firms’ innovativeness and readiness of firms for digital transition [100–102]; sustainability transition and the effects of firms’ environmental behaviour on innovativeness [103]; social, technological, environmental, and managerial aspects contributing to the creation of sustainable and smart cities and regions triggering firms’ innovation [104, 105]; and complementarities between technology transfer activities and different channels of public support for innovation [17].
